# CD56^bri^CD38^+^ as a novel neutrophil-specific marker in chronic myeloid leukemia

**DOI:** 10.1016/j.heliyon.2024.e39465

**Published:** 2024-10-24

**Authors:** Panpan Huang, Cuiping Zhang, Aimei Zhang, Ju Mao, Gan Liu, Chaojie Hu, Huaiping Zhu

**Affiliations:** a Department of Laboratory Medicine, The First Affiliated Hospital of USTC, Division of Life Sciences and Medicine, University of Science and Technology of China, Hefei, Anhui, PR China; b Core Unit of National Clinical Research Center for Laboratory Medicine, Hefei, PR China

**Keywords:** Flow cytometry, Chronic myeloid leukemia, Immunophenotype, CD56, CD38

## Abstract

**Background:**

Chronic myeloid leukemia (CML) is classified as a subtype of myeloproliferative neoplasm, and bone marrow flow cytometry does not reveal any specific immunophenotype. Interestingly, aberrant expression of cluster of differentiation (CD) markers such as CD56 and CD38 has been observed on neutrophils in CML patients. Therefore, we investigated the abnormal expression of CD56 and CD38 in CML neutrophils to explore their diagnostic value in identifying CML through flow cytometry.

**Methods:**

We have developed a multi-parameter flow cytometry assay to identify aberrant immunophenotypes in CML neutrophils among bone marrow nucleated cells.

**Results:**

Compared to healthy donors and patients with a reactive neutrophilia or other hematological malignancies, the percentage of CD56^bri^CD38^+^ neutrophil subsets in CML patients exhibits a distinctive increase (cut-off value, 2.0 %). The specificity and sensitivity associated with the learning cohort (168 samples) were 90.8 % and 84.9 %, respectively, while in the validation cohort (194 samples), they were 90.7 % and 84.7 %. The accumulation of CD56^bri^CD38^+^ neutrophil subsets, which demonstrate are abnormal characteristics, is independent of the neutrophil count, BCR/ABL1 fusions and risk stratification but associated with blast cells immunophenotype. Moreover, this increase disappears in CML patients after treatment with tyrosine kinase inhibitors when the curative effect was satisfactory.

**Conclusions:**

We conclude that an increase in the proportion of CD56^bri^CD38^+^ neutrophil subsets exceeding 2.0 % of total neutrophils serves as a highly sensitive and specific flow cytometry marker, enabling rapid and accurate identification of CML.

## Highlights

• CD56^bri^CD38^+^ neutrophils increase significantly in CML.
• Exceeding the cut-off value can identify CML with high specificity and sensitivity.
• CD56^bri^CD38^+^ neutrophils possess an immature abnormal immunophenotype.
• CD56^bri^CD38^+^ neutrophils decrease immediately in CML satisfied with TKI treatment.

## 1 Introduction

Chronic myeloid leukemia (CML) is a type of myeloproliferative neoplasm characterized by the presence of BCR/ABL1 fusions, resulting from a genetic alteration known as t(9; 22)(q34; q11) [1,2]. The majority of patients are diagnosed during the chronic phase, which is typically marked by an elevation in peripheral blood and bone marrow leukocytes, predominantly neutrophils, along with occasional eosinophils or basophils [3,4]. Morphological examination reveals that both peripheral blood and bone marrow cells primarily consist of neutrophils ranging from myelocyte to rod-shaped nuclei stages; however, flow cytometry does not exhibit any specific immunophenotype [4].

CML was once recognized by Bethesda consensus as one of the diseases that flow cytometry is not suitable for diagnosis, mainly due to the lack characteristic in flow cytometry [5]. The identification of mature neutrophils for CML diagnosis through flow cytometry is challenging, primarily because their immunophenotype closely resembles normal neutrophils. However, apart from expressing cluster of differentiation (CD) markers typical of normal neutrophils such as CD13, CD16, CD11B, CD10, and CD38 [6], CML neutrophils also exhibit abnormal expression of additional CD markers like CD56 [7]. Therefore, these aberrant immunophenotypes are expected to serve as specific indicators of CML using flow cytometry.

CD56 is a membrane glycoprotein expressed in neural and muscular tissues, involved in homotypic adhesion interactions [8]. It is also observed on NK lymphocytes and regulates their cytotoxicity [9]. Aberrant expression of CD56 has been identified in various hematological malignancies such as multiple myeloma [10], chronic myelomonocytic leukemia [11], CML [7], and plays a crucial role in the diagnosis of acute myeloid leukemia as well as minimal residual disease monitoring [12,13]. Similarly, CD38 is a cell surface glycoprotein that catalyzes cyclic adenosine diphosphate ribose synthesis and degradation [14]. It exhibits widespread expression with varying degrees across different lineages of cells [15] and serves as an indicator for cellular activation [16].

In this study, we conducted a comprehensive analysis of the expression patterns of CD56 and CD38 on neutrophils in CML and demonstrated that the increased proportion of CD56^bri^CD38^+^ neutrophil subsets can serve as a specific marker for rapid and accurate recognition of CML using flow cytometry. Interestingly, in chronic myeloid leukemia (CML) patients who exhibited satisfactory response to tyrosine kinase inhibitor treatment, a proportion of CD56^bri^CD38^+^ neutrophil subsets returned to their baseline levels.

## 2 Materials and methods

### 2.1 Patient samples

All patients were treated at our institution and underwent bone marrow or peripheral blood examinations according to medical advice. All bone marrow and/or peripheral blood samples were obtained with informed consent. All patients included in the study were based solely on their diagnosis, regardless of chronic myeloid leukemia (CML) stage. The learning cohort consisted of patients diagnosed with CML according to WHO criteria (n = 53), healthy donors (n = 6), subjects with non-malignant hematological diseases (n = 35), patients with reactive leukocytosis (n = 33), and patients without CML, including acute myeloid leukemia (AML) cases (n = 9), myeloproliferative neoplasms (MPN) cases (n = 24), chronic myelomonocytic leukemia (CMML) cases (n = 3), myelodysplastic syndromes (MDS) cases (n = 3), and B-cell acute lymphoblastic leukemia (B-ALL) cases (n = 2). CML patients were enrolled in a non-interventional study conducted from 2020 to 2021. A validation cohort included CML patients (n = 52) from 2021 to 2023, subjects with non-malignant hematological diseases (n = 45), patients with reactive leukocytosis (n = 49), and patients without CML, including AML (n = 9), MPN (n = 32), CMML (n = 4), and MDS (n = 3). Please refer to Table 1 for detailed information. This study was approved by the Ethics Committee of The First Affiliated Hospital of USTC, and performed in accordance with Declaration of Helsinki.

**Table 1:** Clinical characteristics of patients in learning and validation cohorts.

Parameter	Learning cohort					Validation cohort			
Groups	CML	HD-Co	Non-HM	Non-CML	Reactive	CML	Non-HM	Non-CML	Reactive
Cases	53	6	35	41	33	52	45	48	49
Age (median, range)	50(16–93)	42.5(21–58)	58(19–80)	64(20–90)	56(16–75)	49.5(20–92)	57(18–80)	62.5(16–85)	55(14–85)
Gender (number)									
Male	34	3	15	24	18	34	21	28	25
Female	19	3	20	17	15	18	24	20	24
WBC ∗10^9/L (median, range)	145.5(16.9–548.3)	5.5(3.9–9.0)	6.0(3.3–9.3)	16.3(1.6–230.5)	13.4(9.6–34.6)	142.7(22.6–457.6)	5.5(2.8–8.3)	14.4(1.2–195.9)	13.4(9.8–38.8)
NEC ∗10^9/L (median, range)	120.0(11.4–483.0)	2.7(2.0–6.3)	3.4(1.3–7.0)	8.6(0.3–205.4)	11.0(3.4–32.4)	121.0(17.9–388.5)	3.3(0.7–6.0)	11.5(0.2–130.4)	12.1(6.7–36.2)
CML stages (chronic/accelerated/blast phase)	52/1/0					50/2/0			
BCR-ABL1 (P190/P210/P230)	1/0/52					0/0/52			
BCR/ABL1 (IS)(statistics)	31					23			
% (median, range)	69.7(9.4–197.0)					54.1(14.3–178.7)			
Sokal score (statistics, low/medium/high risk)	34(10/13/11)					26(12/7/7)			

### 2.2 Multi-fluorochrome staining and analysis of bone marrow

Cell staining procedures are performed according to standard operating procedures [17]. Bone marrow samples are prepared and labeled with specific antibodies. All fluorescence-labeled antibodies and panels are shown in Supplementary Table 1. All labeled specimens were collected using BD Canto (II or Plus) flow cytometers (BD Biosciences), ensuring the acquisition of a minimum of 100,000 neutrophils. Instrument settings were harmonized across all devices. Following membrane surface antigen staining, 7-AAD and Annexin V (The apoptosis kit was provided by Wuhan Elabscience Biotechnology Co., Ltd) staining were conducted without red blood cell lysis treatment. The acquired flow cytometry data in FCS format will be centrally analyzed in a blinded manner using Kaluza Analysis Software (Beckman Coulter) to generate scatter plots or density plots. A simple neutrophil gating strategy was referenced for accurate analysis [18].

### 2.3 Detection of BCR/ABL1 fusion

The BCR/ABL1 fusion quantitative detection kit (Shanghai Yuanqi Biomedical Technology Co., Ltd.) was utilized for the assessment of BCR-ABL1 transcripts [19]. All samples from the patient group, control group (comprising negative and positive controls), reference group, and internal reference genome (including the internal reference gene reaction group and its reference group) were amplified using an ABI 7500 fluorescence PCR detector (Thermo Fisher Scientific). Subsequently, baseline and threshold values were determined prior to result analysis. Ultimately, the outcomes were reported in accordance with international standardization.

### 2.4 Statistical analysis

The statistical analysis and plotting were performed using Graphpad Prism 8.0.3 software. The non-parametric Kruskal-Wallis test was employed to compare distributions among groups. Receiver Operator Characteristic (ROC) curves, representing the relationship between sensitivity and specificity, were compared using a nonparametric approach. A cutoff value was estimated in the training cohort by maximizing the Younden index (J = Sensitivity + Specificity −1). The classification performance of the estimated cutoff value was evaluated in all patients and compared to the basophil percentage cutoff of 1.0 % [20]. The ordinary one-way ANOVA was utilized to compare means among groups. Simple linear regression analysis was conducted for comparison purposes. Statistical significance was defined as *P* < 0.05.

## 3 Results

### 3.1 Flow cytometry identification of neutrophils

**Fig. 1:**
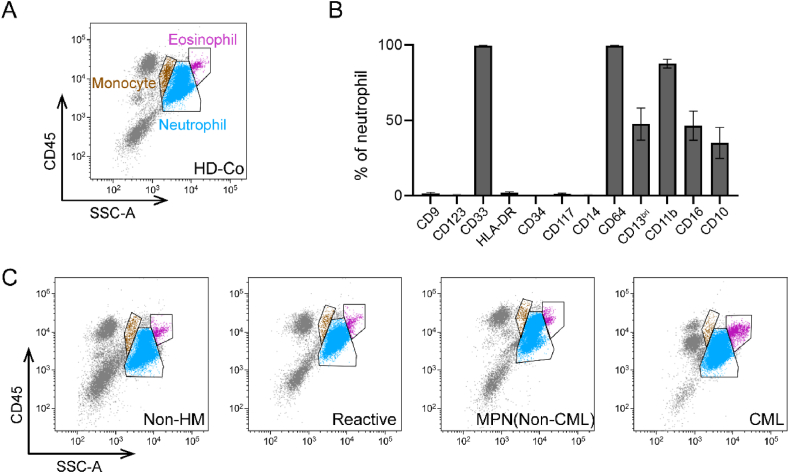
**Immunophenotype of neutrophils in bone marrow (BM).** A. Normal immunophenotype of neutrophils in BM from healthy donors (HD-Co). CD45/SSC-A dot plot gated on Fig. S1C. B. Percentage of cluster of differentiation (CD) markers expression (gated on neutrophils). C. CD45/SSC-A dot plots were obtained from patients with non-hematological malignancies (Non-HM), reactive neutrophilia (Reactive), myeloproliferative neoplasm without chronic myeloid leukemia (Non-CML), and chronic myeloid leukemia (CML).

The neutrophils in bone marrow from healthy donors were analyzed by flow cytometry. A continuous gating strategy (Supplementary Fig. 1) was employed to identify the neutrophil subset in the CD45/SSC-A dot plot (Fig. 1A) [18]. To ensure the reliability of the gating, we assessed a series of CD molecules (Fig. 1B), and successfully distinguished neutrophils from eosinophils and monocytes based on their immunophenotype in the CD45/SSC-A dot plot. By applying the same gating strategy for different patients and excluding potential interference caused by CD34^+^ and/or CD117^+^ blasts (Supplementary Fig. 1E), efficient identification of the neutrophil population was achieved (Fig. 1C). This approach offers a rapid and effective method for gating neutrophils.

### 3.2 A CML signature defined by CD56^bri^CD38^+^ neutrophil subset

**Fig. 2:**
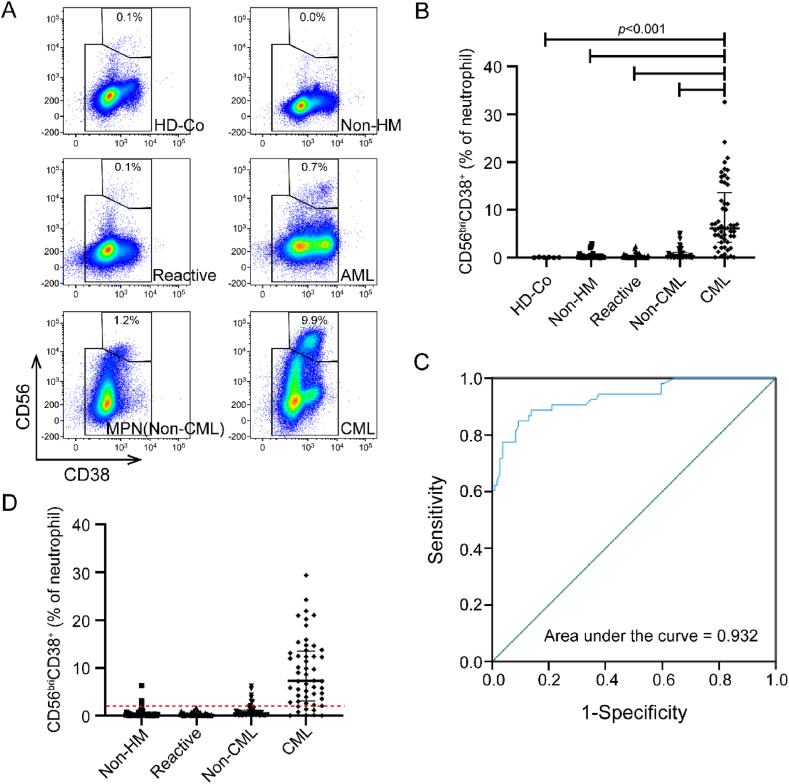
**Elevation in the proportion of CD56**^**bri**^**CD38**^**+**^**neutrophils in chronic myeloid leukemia (CML).** A. Representative flow cytometry analysis comparing CD56/CD38 expression in bone marrow between the learning cohort of CML and healthy donors (HD-Co), non-hematological malignancies (Non-HM), reactive neutrophilia (Reactive), acute myeloid leukemia (AML), and MPN (Non-CML) groups. B. The percentage of CD56^bri^CD38^+^ neutrophils in learning cohort of CML compared to other groups. Median with interquartile range is shown. C. Receiver operating characteristic (ROC) curve analysis of diagnostic sensitivity and specificity of CD56^bri^CD38^+^ neutrophils percentage in bone marrow, established on the learning cohort. D. The percentage of CD56^bri^CD38^+^ neutrophils in a validation cohort of CML compared to Non-HM, Reactive, and Non-CML. Median with interquartile range is shown.

The subset of CD56^bri^CD38^+^ neutrophils either showed absence or occurred at a minimal proportion in groups consisting of healthy donors (HD-Co), individuals with non-hematological malignancies (Non-HM), those with reactive leukocytosis (Reactive), and patients with hematological malignancies (Non-CML). However, it was prominently observed in the chronic myeloid leukemia (CML) patient group (Fig. 2A). To investigate whether the increased proportion of CD56^bri^CD38^+^ neutrophil subset is a distinct characteristic of CML bone marrow, we established a learning cohort for analysis. Within the neutrophil population in the bone marrow, the median percentage of CD56^bri^CD38^+^ neutrophils was 0.1 % (range: 0.0–0.1 %) in HD-Co and 0.2 % (range: 0.0–2.4 %) in Reactive (Fig. 2B). No significant difference was observed between these two groups. The median proportion of CD56^bri^CD38^+^ neutrophils was 0.1 % (range: 0.0–3.0 %) in subjects with Non-HM and 0.2 % (range: 0.0–5.1 %) in subjects with Non-CML (Fig. 2B). In a learning cohort of 53 CML patients, the median percentage of CD56^bri^CD38^+^ neutrophils was significantly higher than other groups (*P* < 0.0001, Kruskal-Wallis test), reaching 6.1 % (range: 0.1–32.5 %, Fig. 2B).

In some cases of CML (n = 7), the CD56^bri^CD38^+^ neutrophils were simultaneously detected in both peripheral blood and bone marrow, with these cells still present in the peripheral blood. Although the distribution of CD56^bri^CD38^+^ neutrophils in peripheral blood and bone marrow exhibited some variation, the observed difference did not reach statistical significance (*P* > 0.05, Kolmogorov-Smirnov test) (Supplementary Fig. 2).

### 3.3 CD56^bri^CD38^+^ neutrophils percentage as a sensitive and specific tool for CML identification

To further investigate the potential auxiliary diagnostic utility of increased CD56^bri^CD38^+^ neutrophils in CML, we conducted a receiver operating characteristic (ROC) analysis using data from the learning cohort. The area under the ROC curve was determined to be 0.932 (95 % Wald confidence limits: 0.888–0.976, Fig. 2C), suggesting that the percentage of CD56^bri^CD38^+^ neutrophils could serve as a discriminatory marker for distinguishing CML from other diseases. The Younden index was defined based on the ROC curve of the learning cohort. The maximum value of this index was utilized as the criterion to determine the optimal cut-off value for CD56^bri^CD38^+^ neutrophils percentage, which was found to be 2.0 %. At this threshold, the specificity and sensitivity were determined to be 90.8 % and 84.9 %, respectively. This cut-off value was further validated in an independent cohort consisting of 52 cases (Fig. 2D), where a specificity of 90.7 % and a sensitivity of 84.7 % were achieved when using a cut-off greater than 2.0 %. Notably, the median percentage of CD56^bri^CD38^+^ neutrophils in validation cohort was 0.2 % (range: 0.0–6.3 %) in Non-HM, 0.2 % (range: 0.0–1.6 %) in Reactive, 0.4 % (range: 0.0–6.2 %) in Non-CML and 7.3 % (range: 0.0–29.4 %) in CML (Fig. 2D).

### 3.4 Comparison of positive rates between CD56^bri^CD38^+^ neutrophils and basophils

**Fig. 3:**
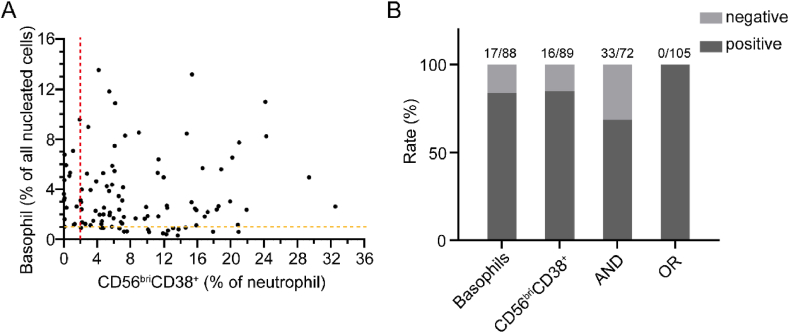
**Comparison of positive rates between CD56**^**bri**^**CD38**^**+**^**neutrophils and basophils.** A. Distribution between the percentage of basophils and CD56^bri^CD38^+^ neutrophils in identifying CML using threshold lines at 1 % for basophils (orange dashed line) and 2 % for CD56^bri^CD38^+^ neutrophils (red dashed line). B. Comparison sensitivity of identifying CML between the positive for CD56^bri^CD38^+^ neutrophils (>2.0 %) and/or positive for basophils (>1.0 %) percentage of basophils. The “AND” group means the positive for CD56^bri^CD38^+^ neutrophils and positive for basophils, and “OR” means there are only one positive.

The percentage of basophils in bone marrow serves as a highly sensitive indicator for the identification of CML, necessitating the utilization of flow cytometry to detect this parameter [21] (Fig. S3). In our cohort of CML patients (n = 105), the sensitivity for identifying CML was 83.8 % (n = 88), based on the criterion of an elevated basophil count exceeding 1.0 %. Furthermore, we observed that an increased percentage of CD56^bri^CD38^+^ neutrophils using a cut-off greater than 2.0 % exhibited a sensitivity for CML of 84.8 % (n = 89), which was slightly higher than that achieved by assessing basophils alone (83.8 %) (Fig. 3A and B). However, this difference did not reach statistical significance (*P* > 0.05, chi-square test). Interestingly, in all patients with CML, there were no instances where both basophils and CD56^bri^CD38^+^ neutrophils percentages fell below the threshold (Fig. 3A and B). It is noteworthy that these two parameters can complement each other for bone marrow flow cytometry-based identification of CML. We found that the positive for CD56^bri^CD38^+^ neutrophils (>2.0 %) or positive for basophils (>1.0 %) can improve the sensitivity of CML to 100.0 %, and this difference in sensitivity is statistically significant (*P* < 0.01, chi-square test) (Fig. 3B).

### 3.5 Abnormal development of CD56^bri^CD38^+^ neutrophils

**Fig. 4:**
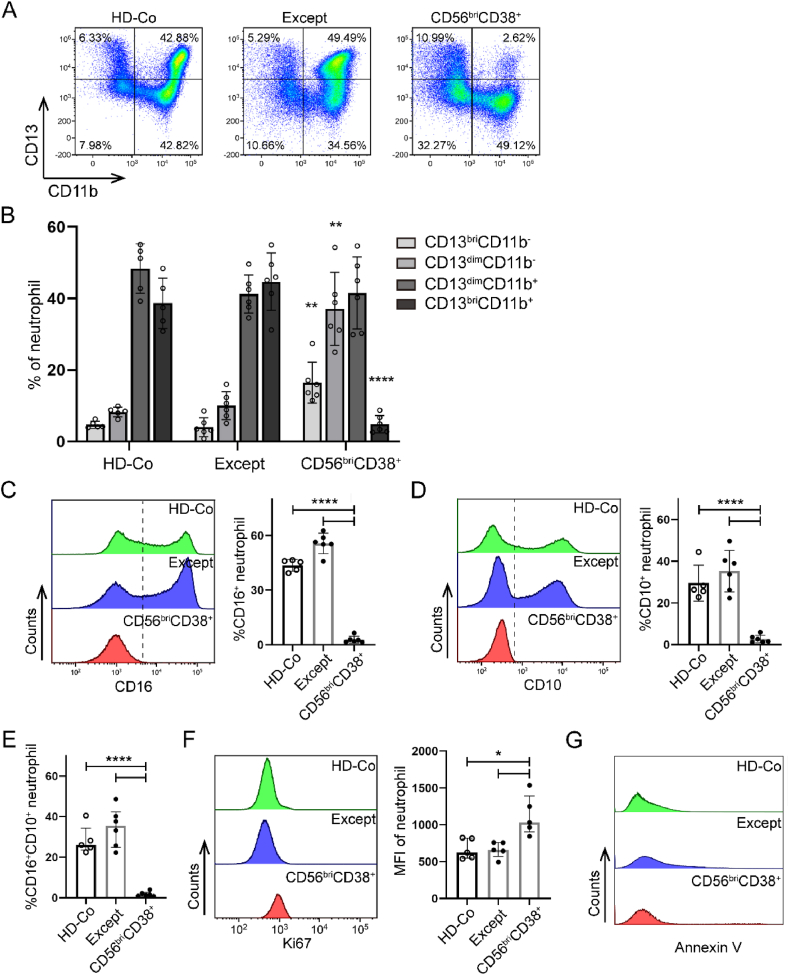
**CD56**^**bri**^**CD38**^**+**^**neutrophils exhibit aberrant immunophenotype, cell proliferation characteristics.** A. Multicolor flow cytometry analysis of CD13 and CD11b between CD56^bri^CD38^+^ neutrophils from CML, neutrophils from HD-Co and neutrophils except CD56^bri^CD38^+^ (Except) from CML. Percentage of each neutrophil subset is indicated. B. The proportion of CD13^bri^CD11b^−^, CD13^dim^CD11b^−^, CD13^dim^CD11b^+^, and CD13^bri^CD11b^+^ subsets in CD56^bri^CD38^+^ neutrophils from CML (n = 6) compared to that in neutrophils from HD-Co (n = 5) and Except (n = 6). Mean values with standard deviation are presented. C and D showed the percentages of CD16^+^ and CD10^+^ neutrophils in CD56^bri^CD38^+^ compared to other groups, respectively. Mean values with standard deviation are shown. E showed the percentages of CD16^+^CD10^+^ neutrophils in CD56^bri^CD38^+^ compared to other groups. F. Fow cytometry analysis of the mean fluorescence intensity of Ki67 in CD56^bri^CD38^+^ compared to HD-Co and Except. G showed the percentages of Annexin V^+^ neutrophils in CD56^bri^CD38^+^ compared to other groups.

To characterize the cellular phasing within the CD56^bri^CD38^+^ neutrophil subset, we assessed the expression percentages of CD13 and CD11b [18,22]. In the HD-Co group (n = 5) and neutrophils except CD56^bri^CD38^+^ (Except) from the CML group (n = 6), we observed diverse cell subpopulations, including CD13^bri^CD11b^−^, CD13^dim^CD11b^−^, CD13^dim^CD11b^+^, and CD13^bri^CD11b^+^. Among these subgroups, the CD13^dim^CD11b^+^ and CD13^bri^CD11b^+^ subsets were predominant (Fig. 4A and B). However, the proportions of cells within the CD56^bri^CD38^+^ neutrophil subpopulations (n = 6) exhibited substantial reconstitution. Specifically, there was a significant increase in the proportions of CD13^bri^CD11b^−^ and CD13^dim^CD11b^−^ cells compared to the HD-Co and Except groups, while the proportion of CD13^bri^CD11b^+^ cells was significantly decreased. The differences in proportions were statistically significant (*P* < 0.01, one-way ANOVA for increases; *P* < 0.0001, one-way ANOVA for decreases) (Fig. 4A and B). Moreover, to elucidate the developmental stage of CD56^bri^CD38^+^ neutrophils, we examined the expression of CD16 and CD10 [18]. Neutrophils from the HD-Co and Except groups displayed partial expression of CD16, whereas CD56^bri^CD38^+^ neutrophils exhibited almost negligible expression of CD16 (Fig. 4C). A similar pattern was observed for CD10 expression (Fig. 4D), and these expression disparities of CD16 and CD10 were statistically significant (*P* < 0.0001, one-way ANOVA) (Fig. 4E). Hence, CD56^bri^CD38^+^ neutrophils represent a population of immature or naive granulocytes with dyssynchronous pattern of CD13, CD11b and CD16 expression.

To investigate the proliferative capacity of CD56^bri^CD38^+^ neutrophils, Ki67 expression was quantified using flow cytometry. The mean fluorescence intensity of Ki67 in CD56^bri^CD38^+^ neutrophils (n = 5) exhibited a significantly higher level compared to that both in HD-Co (n = 5) and Except (n = 5) groups (Fig. 4F), with statistical significance observed (*P* < 0.05, one-way ANOVA). Additionally, the expression of Annexin V was examined to assess the apoptotic ability of CD56^bri^CD38^+^ neutrophils, and no significant differences were observed among the groups (Fig. 4G). Taken together, these findings suggest that CD56^bri^CD38^+^ neutrophils represent a population of immature or naive granulocytes with an increased capacity for proliferation.

### 3.6 The proportion of CD56^bri^CD38^+^ neutrophils was dependent of blast cell immunophenotype

**Fig. 5:**
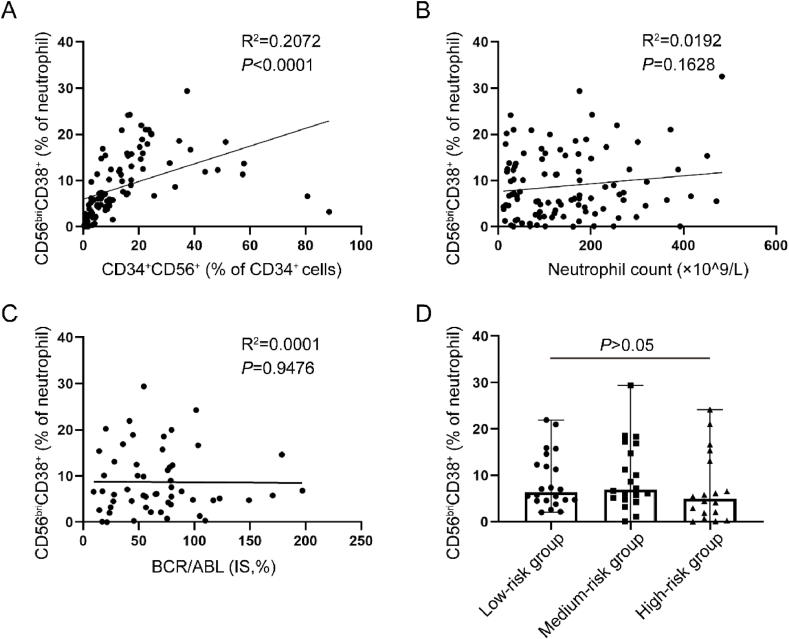
**The percentage of CD56**^**bri**^**CD38**^**+**^**neutrophils was independent of neutrophil count, risk stratification and BCR/ABL1 fusion gene but correlated with blast cells immunophenotype.** A. Correlation of the percentage of CD56^bri^CD38^+^ neutrophils with percentage of CD56^+^ form CD34^+^ cells. B. Correlation of the percentage of CD56^bri^CD38^+^ neutrophils with neutrophil count. C. Correlation of the percentage of CD56^bri^CD38^+^ neutrophils determined by flow cytometry with the BCR/ABL1 (IS, %). D. Correlation of the percentage of CD56^bri^CD38^+^ neutrophils determined by flow cytometry with different risk groups (grouped according to Sokal score).

Given the aberrant immunophenotype of CD56 expression in the blasts [22,23], we sought to investigate whether there is a correlation between the percentage of CD56^bri^CD38^+^ neutrophils and the immunophenotype of the blasts. We found a significant association between the percentage of CD56^bri^CD38^+^ neutrophils and the proportion of CD56^+^ cells within the CD34^+^ cell population (R^2^ = 0.2072, *P* < 0.0001) (Fig. 5A).

The increased neutrophils and the presence of BCR/ABL1 fusion gene are characteristics manifestation of CML. In here, we aimed to investigate whether the proportion of CD56^bri^CD38^+^ neutrophils correlate with both the neutrophil count and BCR/ABL1 (IS, %) levels. Our findings revealed that the percentage of CD56^bri^CD38^+^ neutrophils remained unaffected by variations in both the neutrophil count and BCR/ABL1 fusion gene (Fig. 5B and C). To investigate the potential association between the CD56^bri^CD38^+^ neutrophil subpopulation and prognosis in CML, all CML patients were categorized into three risk groups (low, medium, high) based on their Sokal score [24]. However, no significant differences were observed in the percentage of CD56^bri^CD38^+^ neutrophils among these risk groups (Fig. 5D).

### 3.7 The proportion of CD56^bri^CD38^+^ neutrophils serve as one of the biomarkers for CML blast crisis after treatment

**Fig. 6:**
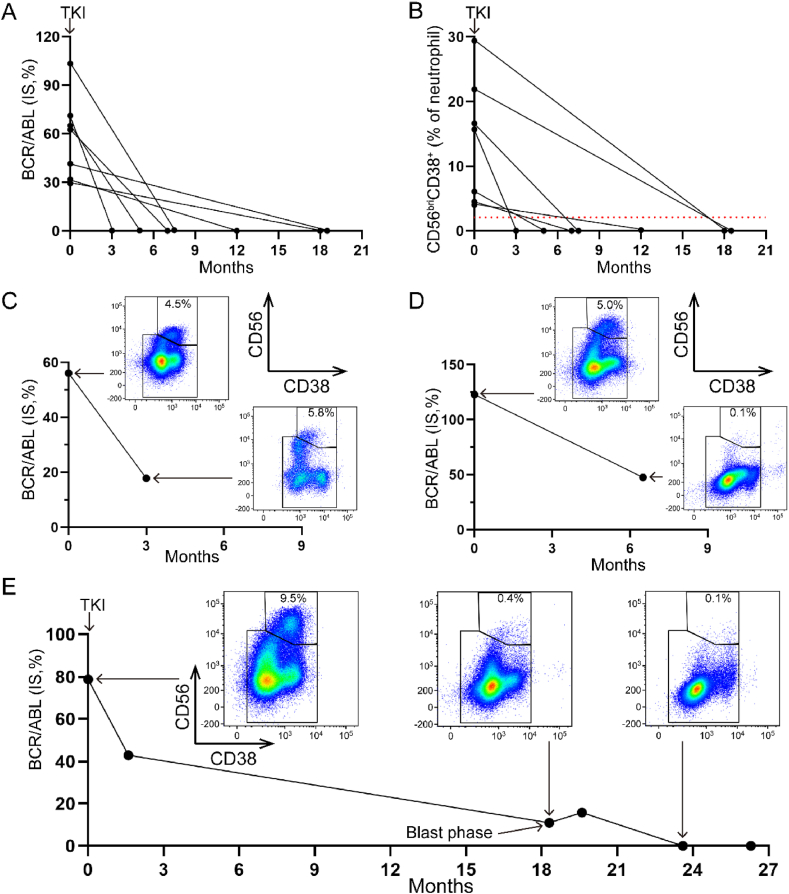
**The changes in percentage of CD56**^**bri**^**CD38**^**+**^**neutrophils after treatment.** A. Patients (n = 7) with BCR/ABL1 fusion gene (IS, %) achieved satisfactory curative effect after tyrosine kinase inhibitor (TKI) treatment. B. CD56^bri^CD38+ neutrophils dropped below the cut-off (2.0 %) value in patients from (A) after treatment. C. The CD56^bri^CD38^+^ neutrophils did not decrease in patients who did not achieve satisfactory curative effect after treatment. D. The CD56^bri^CD38^+^ neutrophils decreased below the cut-off value in patients who did not achieve satisfactory curative effect after treatment. E. BCR/ABL1 fusion gene and CD56^bri^CD38^+^ neutrophils change over time in patients under the blast phase of CML.

Efficacy satisfaction was assessed according to the guidelines outlined in the NCCN for Patients Version 2023 [25]. In the initial cohort of seven patients diagnosed with chronic phase, treatment with tyrosine kinase inhibitor (TKI) showed favorable outcomes, as evidenced by positive responses (Fig. 6A). All patients demonstrated a decrease in the percentage of CD56^bri^CD38^+^ neutrophils to below 2.0 % (Fig. 6B). Disappointingly, patients who expressed dissatisfaction with TKI treatment still exhibited a CD56^bri^CD38^+^ neutrophil percentage above 2.0 % (Fig. 6C). The above indicates that the proportion of CD56^bri^CD38^+^ varies coordinately by the efficacy of the treatment. Notably, CD56^bri^CD38^+^ neutrophils decreased to below the predetermined threshold values even in patients unsatisfied with TKI therapy (Fig. 6D), while flow cytometry-based assessment of minimal/measurable residual disease (MRD) remained positive (data not shown). Consequently, positive BCR/ABL1 (IS) results in these patients were attributed to the presence of MRD-positive blast cells. Furthermore, in a patient with blast phase (Fig. 6E), the initial BCR/ABL1 (IS) level was 78.8 %, accompanied by a CD56^bri^CD38^+^ neutrophil proportion of 9.5 %. Following 18.3 months of treatment with TKIs, the BCR/ABL1 (IS) level decreased to 11.0 %, and the percentage of CD56^bri^CD38^+^ neutrophils reduced to 0.4 %. Subsequently, after undergoing acute leukemia therapy, both BCR/ABL1 (IS) levels and the proportion of CD56^bri^CD38^+^ neutrophils dropped below the designated threshold values. Taken together, the inability to decrease BCR/ABL1 (IS) levels concurrent with declining proportions of CD56^bri^CD38^+^ neutrophils may indicate active proliferation of abnormal blast cells. These findings highlight the potential of monitoring the percentage of CD56^bri^CD38^+^ neutrophils as a valuable marker for blast crisis.

## 4 Discussion

It is well established that CD56 is highly expressed on natural killer (NK) lymphocytes and CD38 on plasma cells, serving as their relatively specific markers. Co-expression of CD56 and CD38 can also be observed in the hematopoietic system; for instance, activated NK cells can express CD38 [26,27], and abnormal plasma cells can exhibit high levels of CD56 [10,28]. These conditions thus define their specific immunophenotype when assessed using flow cytometry. Despite expressing a variety of cluster of differentiation (CD) molecules, neutrophils lack specific markers to identify particular disease conditions. In this study, we investigated the characteristics of CD56^bri^CD38^+^ neutrophils in chronic myeloid leukemia (CML) and assess their potential as auxiliary diagnostic and monitoring tools for the disease.

While flow cytometry has its limitations in the diagnostic realm of CML, its role in CML continues to progress. Current research in this area focuses on identifying abnormal immunophenotypes in CML stem and progenitor cells [29,30]. However, distinguishing CML from other myeloproliferative neoplasms (MPNs) remains a challenge. The specific immunophenotypes of basophils serves as a flow-marker for CML [31], yet this aspect is often overlooked in routine clinical flow cytometry unless basophil counts are elevated. Therefore, there is a persistent need for high specificity and sensitivity methods to accurately identify CML. Notably, the CD markers CD56 and CD38, commonly used in identifying blood cell populations, have proven valuable in diagnosing and treating blood disorders [32,33]. While instances exist of normal neutrophils expressing CD56 [34,35] or CD38 [16,36], our study highlights a distinct elevation in CD56^bri^CD38^+^ neutrophils unique to CML patients, rarely observed in other diseases like MPNs. This observation underscores the specificity and sensitivity of detecting CD56^bri^CD38^+^ neutrophils as a promising auxiliary diagnostic tool for CML, offering utility in both healthy individuals and patients.

For the first time, we have uncovered a specific increase in the proportion of CD56^bri^CD38^+^ neutrophil subset in CML, offering initial insights into the developmental stages of this unique cell population. Normally, bone marrow granulocytes progress through various stages of differentiation according to established patterns [18,21,31]. It is anticipated that bone marrow neutrophils predominantly comprise cells in late infancy and mature stages, characterized by an immunophenotype dominated by CD13^dim^CD11b^+^ and CD13^bri^CD11b^+^ cell populations. Additionally, normal neutrophils express CD16 [37,38] and CD10 [39,40], essential for their functional roles. However, our research reveals that CD56^bri^CD38^+^ neutrophils deviate from this norm, lacking the CD13^+^CD11b^+^ subset and showing minimal expression of CD16 and CD10 markers, thus categorizing them as a distinct immature subset. Moreover, these cells demonstrate heightened proliferative capacity, potentially contributing to the elevated white blood cell count. Overall, the abnormal differentiation and characteristics exhibited by CD56^bri^CD38^+^ neutrophils impede their maturation process, shedding light on the pathogenesis of CML. This subset of neutrophil is expected to be one of the therapeutic targets for CML.

Although tyrosine kinase inhibitors (TKIs) have shown remarkable efficacy in providing long-term relief for the majority of CML patients, a small subgroup continues to face disease progression as a result of TKI treatment failure [41]. As outlined in the NCCN guidelines Version 1.2024 [24], there exists a defined reference range for BCR/ABL1 fusion levels at specified time points post-treatment, which not only serves as a measure of drug effectiveness but also offers insights for tailoring subsequent treatment approaches [41,42]. Our study reveals that in cases where TKIs demonstrate a positive therapeutic response, the population of CD56^bri^CD38^+^ neutrophils diminishes concurrently with the reduction of BCR/ABL1 fusion levels. Conversely, in scenarios marked by inadequate TKI efficacy, fluctuations in the percentage of CD56^bri^CD38^+^ cells do not align with changes in TKI response. This discordance may potentially stem from blast cells, as our analysis solely focuses on neutrophils while overlooking the involvement of blast cells. However, on the other hand, it can be suggested that BCR/ABL1 fusion levels combined with CD56^bri^CD38^+^ neutrophils count can hint the acceleration of blast cells in patients after CML treatment.

In conclusion, the observed elevation in the proportion of CD56^bri^CD38^+^ neutrophil subset represents a distinctive feature of CML neutrophils, thereby offering significant auxiliary diagnostic value for flow cytometry in identifying newly diagnosed CML patients. Consequently, establishing reference cut-off values for peripheral blood becomes imperative to facilitate fast universal screening of individuals with elevated white blood cell counts.

## CRediT authorship contribution statement

**Panpan Huang:** Writing – original draft, Software, Investigation, Formal analysis, Data curation, Conceptualization. **Cuiping Zhang:** Resources, Investigation, Formal analysis, Data curation. **Aimei Zhang:** Resources, Data curation. **Ju Mao:** Resources, Data curation. **Gan Liu:** Visualization. **Chaojie Hu:** Writing – review & editing, Funding acquisition. **Huaiping Zhu:** Funding acquisition, Conceptualization.

## Ethical statement

This study was approved by the Ethics Committee of the First Affiliated Hospital of USTC on July 27, 2023 (No: 2023-RE-248).

## Data availability

The data obtained from this study can be made available upon reasonable request.

## Funding

This study was supported by 10.13039/501100001809National Natural Science Foundation of China (Grant no. 31870897; 82370594; 82070601), Central University Basic Scientific Research Business Expenses Special Funds (Grant no. WK9110000001) and 10.13039/501100017668Anhui Provincial Key Research and Development Plan (Grant no. 1804h08020245).

## Declaration of competing interest

No conflicts of interest to disclose.
