# Supplementary material for: CD56briCD38+ as a novel neutrophil-specific marker in chronic myeloid leukemia
Source: Heliyon. 2024 Oct 24;10(21):e39465. doi: 10.1016/j.heliyon.2024.e39465 (PMC11570290; doi:10.1016/j.heliyon.2024.e39465)
Supplement: Multimedia component 1 [file mmc1.docx]

**Supplementary**


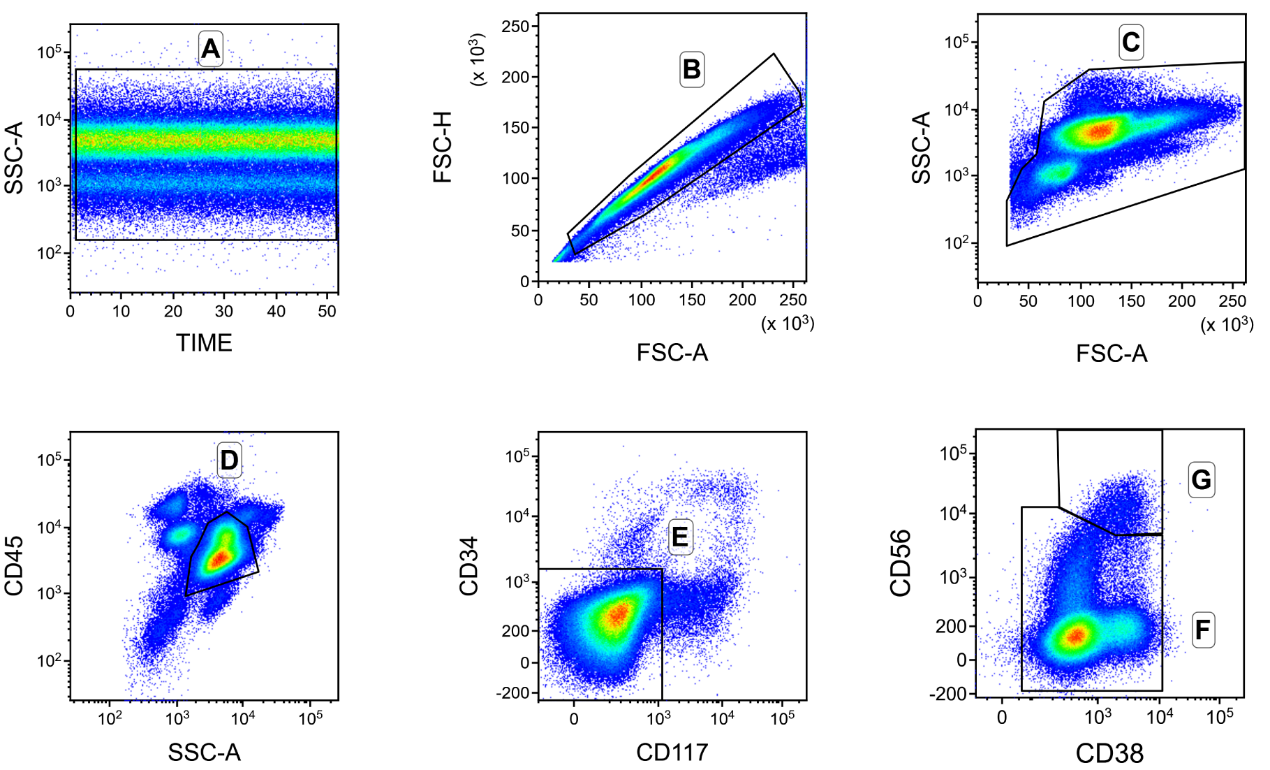


**Figure S1. Neutrophils and CD56^bri^CD38^+^ subset gating strategy.** (A) represents a continuous flow of cells under the control of SSC-A and TIME. Non-adherent cells (B) were excluded using FSC-A/FSC-H parameters. (C) represents the cell that uses FSC-A/SSC-A parameters to remove cell debris. CD45/SSC-A dot plot gated on C to set the neutrophils gate (D). CD34/CD117 dot plot gated on D to remove the blast cells (E). CD56/CD38 dot plot gated on E to separate into two groups of cells (G and F).


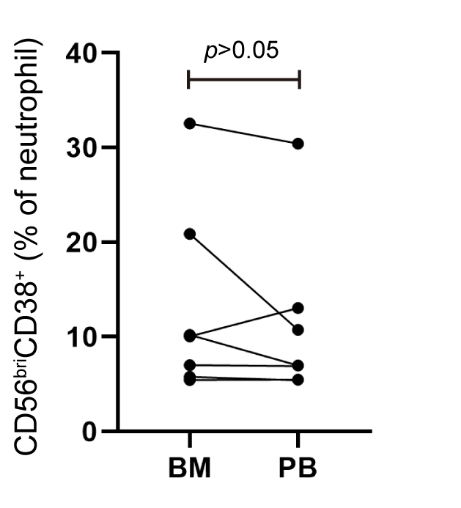


**Figure S2. Comparison of the proportion of CD56^bri^CD38^+^ neutrophils in the bone marrow (BM) and peripheral blood (PB) of patients.**


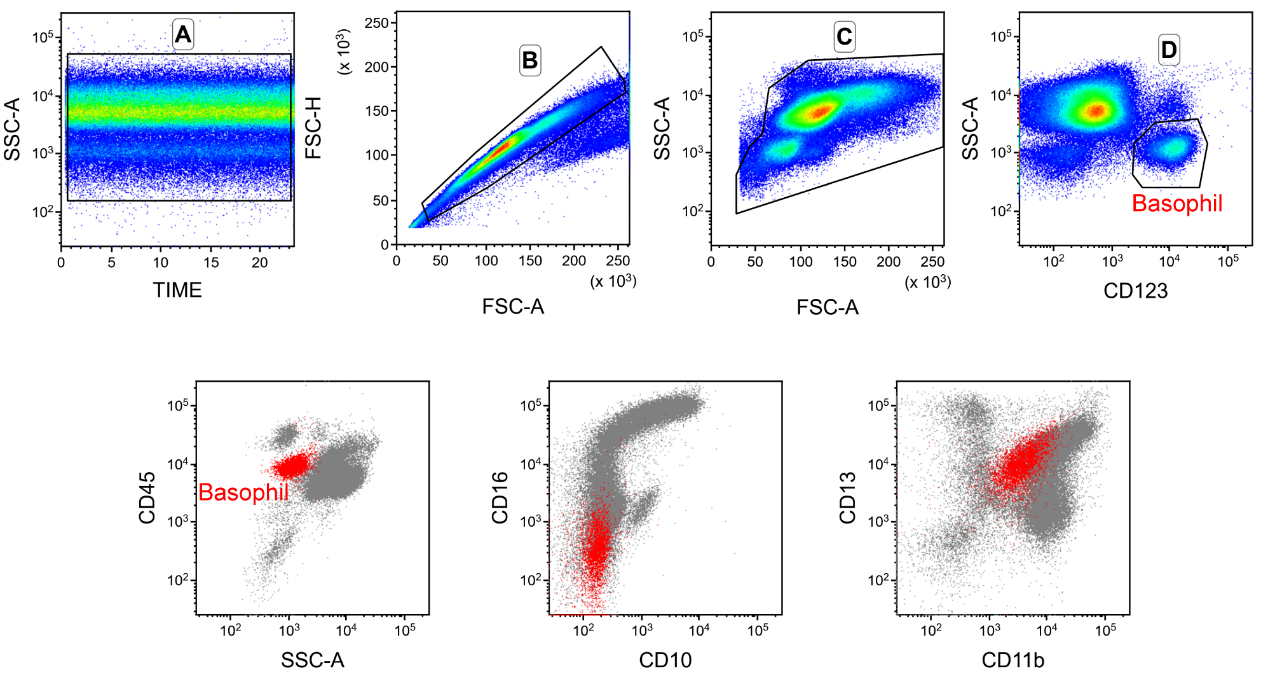


**Figure S3. The basophil gate strategy.** (A), (B) and (C) are equivalent to gate (A), (B) and (C) in Figure S1. Gate D represents medium-sized cells expressing CD123^bri^. Basophils were identified based on their expression of CD45/SSC, CD16/CD10, CD13/CD11B in these plots.
